# A phase II study of FOLFIRI-3 (double infusion of irinotecan combined with LV5FU) after FOLFOX in advanced colorectal cancer patients

**DOI:** 10.1038/sj.bjc.6603095

**Published:** 2006-04-11

**Authors:** M Mabro, P Artru, T André, M Flesch, F Maindrault-Goebel, B Landi, G Lledo, A Plantade, C Louvet, A de Gramont

**Affiliations:** 1 Department of Medical Oncology, Hôpital Foch, 40 rue Worth, 92151 Suresnes Cedex, France; 2 Department of Medical Oncology, Clinique Saint-Jean, Lyon, France; 3 Department of Medical Oncology, Hôpital Tenon, Paris, France; 4 Department of Medicine, Hôpital de Dijon, Dijon, France; 5 Department of Medical Oncology, Hôpital Saint-Antoine, Paris, France; 6 Department of Medical Oncology, Hôpital Européen Georges Pompidou, Paris, France

**Keywords:** colorectal cancer, combination chemotherapy, irinotecan, 5-fluorouracil, leucovorin, phase II study

## Abstract

In advanced colorectal cancer previously treated with oxaliplatin, efficacy of irinotecan-based chemotherapy is poor and the best regimen is not defined. We designed FOLFIRI-3 and conducted a phase II study to establish its efficacy and safety in advanced colorectal cancer patients previously treated with FOLFOX. FOLFIRI-3 consisted of irinotecan 100 mg m^−2^ as a 60-min infusion on day 1, running concurrently with leucovorin 200 mg m^−2^ as a 2-h infusion on day 1, followed by 46-h continuous infusion of 5-fluorouracil (5FU) 2000 mg m^−2^, and irinotecan 100 mg m^−2^ repeated on day 3, at the end of the 5FU infusion, every 2 weeks. Sixty-five patients entered the study. The intent-to-treat objective response rate was 23% (95% CI 13–33%). Disease was stable in 37% of patients, progressed in 26% and was not assessable in 14%. From the start of FOLFIRI-3, median progression-free survival was 4.7 months and median survival 10.5 months. Main toxicities (% of patients) were grade 3–4 diarrhoea 23% and grade 4 neutropenia 11%. FOLFIRI-3 is a promising regimen achieving high response rate and progression-free survival in patients previously treated with FOLFOX with a moderate toxicity.

Colorectal cancer is the one of the commonest cancers worldwide and remains a leading cause of mortality. For many years the main treatment for metastatic colorectal cancer consisted of 5-fluorouracil (5FU) modulated by leucovorin (LV). Since 1990, tremendous progresses have been made through the availability of several new drugs, such as oxaliplatin and irinotecan (Cunningham *et al*, 1998; Rougier *et al*, 1998; De Gramont *et al*, 2000; Douillard *et al*, 2000; Saltz *et al*, 2000; Grothey *et al*, 2002; Goldberg *et al*, 2004).

Irinotecan has definite activity against advanced metastatic colorectal cancer both in chemotherapy-naive patients and after 5FU failure (Cunningham *et al*, 1998; Rougier *et al*, 1998; Douillard *et al*, 2000; Saltz *et al*, 2000). Two randomised studies in patients with 5FU bolus-resistant metastatic colorectal cancer established the superiority of irinotecan over best supportive care (Cunningham *et al*, 1998) or 5FU continuous-infusion regimens (Rougier *et al*, 1998). Later, two large multicentre randomised studies established the superiority of irinotecan combined with 5FU and LV over 5FU–LV as first-line treatment of metastatic colorectal cancer (Douillard *et al*, 2000; Saltz *et al*, 2000). Two additional randomised studies demonstrated the superiority of oxaliplatin combined with LV5FU2 (FOLFOX4) over 5FU and LV as front therapy (De Gramont *et al*, 2000; Grothey *et al*, 2002). More recently, in a large phase III study, FOLFOX4 achieved better response rate, progression-free survival and overall survival than the irinotecan-based IFL regimen as first-line treatment of metastatic colorectal cancer (Goldberg *et al*, 2004). However, few data are available about irinotecan-based chemotherapy in patients previously treated with FOLFOX. Thus, the FOLFIRI regimen achieved a 5% response rate in pretreated patients (André *et al*, 1999; Tournigand *et al*, 2004).

*In vitro* studies suggested that the synergy between irinotecan and 5FU is sequence dependent, with a higher cytotoxicity when irinotecan is administered before 5FU (Guichard *et al*, 1997; Mullany *et al*, 1998; Mans *et al*, 1999). Clinical data are less documented. In a phase II randomised study evaluating the sequence effect of irinotecan and 5FU, toxicities were affected by the sequence of administration, with a worsened tolerability when 5FU was followed by irinotecan (Falcone *et al*, 2001). These results suggested that cytotoxicity is higher when irinotecan is administered after 5FU (Falcone *et al*, 2001). Thus, we previously designed the FOLFIRI-2 regimen consisting of irinotecan 180 mg m^−2^ combined with a simplified LV5FU regimen, with irinotecan administered at the end of the 5FU infusion (Mabro *et al*, 2003). In this phase II study of patients with heavily pretreated colorectal cancer, efficacy was encouraging, with a 17% confirmed response rate and 4.1 months progression-free survival, but toxicity was high.

Thus, irinotecan-based chemotherapy after FOLFOX needs to be improved and the best schedule is not yet established. We designed a new regimen, FOLFIRI-3, in which irinotecan is administered as two infusions: half-dose before 5FU and half-dose at the end of the 5FU infusion, and conducted this multicentre phase II study to evaluate the efficacy of the FOLFIRI-3 regimen in advanced colorectal cancer patients previously treated with FOLFOX regimen.

## PATIENTS AND METHODS

This study was an open multicentre phase II study conducted from June 2001 to December 2002. The study fulfilled the Clinical Good Practice Guidelines and was approved by the Ethics Committee of La Pitié – Salpêtrière Hospital, Paris, France. Written informed consent was obtained from all patients prior to study entry.

The main end point of the study was progression-free survival. The other study parameters were: response rate, overall survival and toxicity.

### Inclusion criteria

Patients were required to be 18–80 years old, and to have metastatic adenocarcinoma of the colon or the rectum histologically proven and previously treated with a FOLFOX regimen. An interval of at least 2 weeks must have elapsed since prior treatment. Other eligibility criteria were: no central nervous system metastases, WHO performance status 0–2, initial evaluation ⩽2 weeks before inclusion by computed tomography scan prior to initiation of therapy and feasibility of regular follow-up. Required laboratory parameters included: neutrophil count ⩾1500 *μ*l^−1^, platelet count ⩾100 000 *μ*l^−1^, serum alkaline phosphatase <3 times the upper limit of normal (ULN) and bilirubin <1.5 times ULN.

### Treatment administration

**Figure 1:**
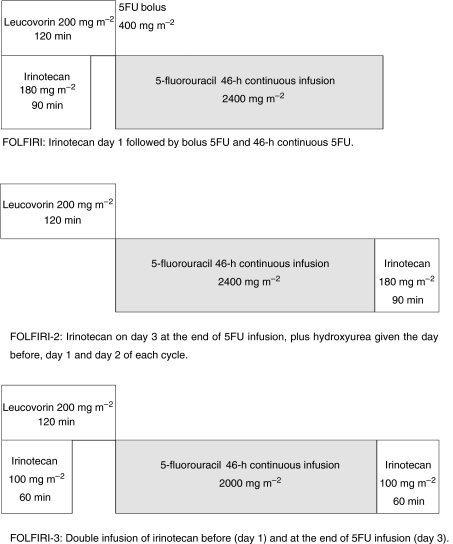
Design of FOLFIRI (André *et al*, 1999), FOLFIRI-2 (Mabro *et al*, 2003) and FOLFIRI-3 regimens; cycles are repeated every 2 weeks.

FOLFIRI-3 regimen was given every 14 days as follows: on day 1, irinotecan 100 mg m^−2^ as a 1-h infusion, running concurrently with LV 200 mg m^−2^ as a 2-h infusion via a Y-connector, followed by 5FU 2000 mg m^−2^ as a 46-h infusion using a disposable elastomeric pump. On day 3, irinotecan 100 mg m^−2^ as a 1-h infusion was repeated, at the end of the 5FU infusion (Figure 1). In the absence of grade 2 toxicity after two cycles, the dose of irinotecan was to be increased from 100 to 120 mg m^−2^ at days 1 and 3. Treatment was continued until disease progression or limiting toxicity.

Blood tests and clinical evaluation were performed every 2 weeks, prior to treatment. Chemotherapy could be administered if neutrophil count was ⩾1500 *μ*l^−1^, platelet count ⩾100 000 *μ*l^−1^ and if clinical toxicity was resolved or <grade 2. In case of grade >2 neutropenia, thrombocytopenia or diarrhoea toxicity, the dose of irinotecan was to be reduced from 100 to 80 mg m^−2^. If neutropenia, thrombocytopenia or diarrhoea >grade 2 persisted at this latter dose, chemotherapy was discontinued.

### Supportive care

Specific anti-emetic prophylaxis was left to the investigator's discretion. For patients who experienced an early cholinergic syndrome occurring during or shortly after irinotecan administration, atropine sulphate (0.25 mg) could be given subcutaneously. The use of loperamide was recommended in case of diarrhoea.

### Study parameters

Before each cycle, patients underwent a clinical examination, and blood cells were counted. All toxicities were reported according to the National Cancer Institute-Common Toxicity Criteria (NCI-CTC) (MacDonald *et al*, 1995). Carcinoembryogenic antigen, bilirubin, serum alkaline phosphatases, serum creatinine, lactate dehydrogenase, chest X-ray and CT scans were repeated every 8 weeks (i.e. every four cycles) or earlier in the case of worsening of clinical condition. Tumour response was assessed by RECIST criteria (Therasse *et al*, 2000). Tumour growth control was defined by the proportion of patients with response rate or stable disease.

### Statistical considerations

Based on previous studies, we expected FOLFIRI-3 to achieve a 40% rate of nonprogressive patients at 6 months. According to a binomial distribution point estimate of proportion, with 5% significance and 80% power, 65 patients were to be enrolled. Survival times were calculated from the start of FOLFIRI-3 until death. Time to progression was calculated from the first day of FOLFIRI-3 to the date of progression for all the patients entering the study. Survival curves were obtained using the Kaplan–Meier method (Kaplan and Meier, 1958).

## RESULTS

From 17 June 2001 to 19 December 2002, 65 patients were recruited from nine French institutions. Their characteristics are described in Table 1. All patients had previously been treated with a FOLFOX regimen (combination of oxaliplatin, infusion 5FU and LV), and had experienced disease progression while on FOLFOX (36 patients) or after discontinuation (29 patients). Thirteen patients (20%) had previously been treated with at least two regimens for metastatic purpose before entering the FOLFIRI-3 study.

### Treatment

The median number of treatment cycles administered was 7 (range, 1–20). Six patients (9%) refused to continue after the first or the second course (personal convenience without toxicity >grade 2). In six patients (9%) treatment was stopped because of grade 3 or 4 toxicity. Tumour response was not assessable in nine of those 12 patients. In 39 patients (60%) treatment was stopped because of disease progression after a median of eight courses. In 14 additional patients (21%) chemotherapy was interrupted by investigator after 8–20 cycles, without evidence of disease progression nor severe toxicity.

Dose reductions occurred in 25 patients due to grade 3 toxicities, mainly vomiting, diarrhoea or mucositis. Irinotecan dose was increased according to the design of the study only in 12 patients with a median dose level of 120 mg m^−2^ on days 1 and 3. Grade 3 toxicity occurred only in one of these patients.

### Safety

Side effects were collected for 463 cycles according to the NCI-CTC grade scale. Toxicities are listed in Table 2. Neutropenia reached grade 3/4 in 37% of patients, including three patients (5%) who had one episode of febrile neutropenia. There was one toxic death after the first course of chemotherapy related to a sepsis shock with neutropenia and diarrhoea. Overall, 10 patients (15%) experienced at least one grade 4 toxicity and 20 patients (31%) experienced one grade 3 nonhematological toxicity.

### Survival and objective response

**Figure 2:**
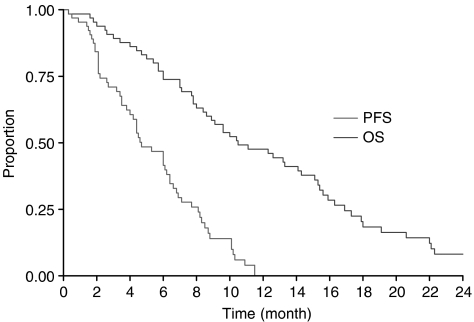
Progression-free survival and overall survival in the FOLFIRI-3 study (65 patients).

Progression free survival for 6 months was 42%. From the start of FOLFIRI-3, median progression-free survival was 20.5 weeks (4.7 months) and median survival was 46 weeks (10.5 months). Survival curves are shown in Figure 2.

Tumour response was assessable in 56 patients and not assessable in nine patients (14%). Response rates were calculated in the intent-to-treat population. Objective response was obtained in 15 patients (1 complete, 14 partial), achieving a 23% (95% CI 13–33%) response rate (15 out of 65). The number of responder patients assessed by RECIST criteria are listed in Table 3. Disease was stable in 24 patients (37%) and progressed in 17 (26%). Tumour growth control was 60% (95% CI 48–72%).

Regarding assessable patients, response rate was 27% (95% CI 15–39%), and 22% in the subset of patients in whom disease progressed while on previous FOLFOX regimen, and 33% in the subset of patients in whom disease progressed after FOLFOX discontinuation.

## DISCUSSION

This multicentre phase II study assessed the efficacy and tolerability of the FOLFIRI-3 regimen, a new combination of irinotecan and LV5FU, in patients with metastatic colorectal cancer previously treated with FOLFOX. With 42% of patients free of progression at 6 months, the main objective of the study was reached.

Several phase II studies (Pitot *et al*, 1997; Rougier *et al*, 1997; Rothenberg *et al*, 1999; Van Cutsem *et al*, 1999) and two large phase III studies (Cunningham *et al*, 1998; Rougier *et al*, 1998) established the efficacy of irinotecan after 5FU failure (Table 4). In phase II studies of irinotecan alone, after 5FU failure response rates were 11–16% (Rougier *et al*, 1997; Rothenberg *et al*, 1999; Van Cutsem *et al*, 1999), and progression-free survival was 3–4 months (Pitot *et al*, 1997; Rougier *et al*, 1997; Rothenberg *et al*, 1999; Van Cutsem *et al*, 1999). In phase III studies of irinotecan after 5FU failure, progression-free survival was 3–4 months (Cunningham *et al*, 1998; Rougier *et al*, 1998; Fuchs *et al*, 2003). Response rate was reported in only one of those studies and was 4.5% (Rougier *et al*, 1998).

The combination of irinotecan with 5FU and LV after 5FU failure was evaluated in three phase II studies. Reported response rates were 11.4–21% (Ducreux *et al*, 1999; Gil-Delgado *et al*, 2001; Rougier *et al*, 2002). As shown in Table 4, results observed with regimens combining irinotecan, 5FU and LV after 5FU failure seem slightly better than those with irinotecan alone, in a cross-study comparison. More recently, a large randomised phase III study was conducted enrolling more than 2000 patients. In this study, the addition of irinotecan to 5FU after 5FU failure achieved better survival (14.8 *vs* 13.9 months from the start of front-line 5FU) and higher response rate (21 *vs* 11%) than irinotecan alone after 5FU failure (Seymour *et al*, 2005). These results encourage the administration of 5FU with irinotecan despite previous 5FU failure.

Efficacy of irinotecan in patients previously treated with FOLFOX is less documented. Results of the FOLFIRI, FOLFIRI-2 and FOLFIRI-3 studies are summarised in Table 4. In a randomised trial recently reported, irinotecan alone achieved a 4% response rate and 2.7 progression-free survival in 94 patients previously treated with 5FU as front line and FOLFOX4 as second line (Rowland *et al*, 2005).

In the present study, the progression-free survival was 4.7 months and the intent-to-treat response rate was 23%, higher than those reported with irinotecan alone, FOLFIRI and FOLFIRI-2 after FOLFOX. In a cross-study comparison, efficacy of FOLFIRI-3 seems slightly better than FOLFIRI-2, as FOLFIRI-2 showed a high toxicity profile. The main FOLFIRI-2 grade 3–4 toxicities were diarrhoea (31%), neutropenia (52%), and febrile neutropenia (14%) (Mabro *et al*, 2003). Toxicity of FOLFIRI-3 was moderate, lower than FOLFIRI-2 study and quite higher than FOLFIRI in pretreated patients (André *et al*, 1999). It is difficult to assess if this increased toxicity is related to the divided dose of irinotecan or to the total dose of irinotecan, which is 10% more than in the FOLFIRI (200 instead of 180 mg m^−2^ per cycle). Differences should be also partly due to the subset of patients. In particular, patients of the FOLFIRI-3 study were less selected and 17% entered the study with a PS 2.

In conclusion, FOLFIRI-3 is a feasible regimen with encouraging response rate and progression-free survival in patients previously treated with FOLFOX. Future studies should evaluate the combination of FOLFIRI-3 with molecular targeted therapies like bevacizumab, cetuximab or new oral anti-REGF molecules.

## Figures and Tables

**Table 1:** Patient characteristics (*n*=65)

	***N***	**%**
Median age: 60 years (range: 30–79)		
		
*Gender*		
Male	32	49
Female	33	51
		
*WHO performance status*		
0	32	49
1	22	34
2	11	17
		
*Primary tumour*		
Colon	49	75
Rectum	16	25
		
*Site of metastases*		
Liver	52	80
Lung	23	35
Peritoneum	9	14
Nodes	11	17
Other	6	9
		
*Involved sites*		
1	36	55
2	23	35
>2	6	9
		
*Prior adjuvant chemotherapy:*	13	20
LV5FU	11	17
FOLFOX	2	3
		
*Number of lines for metastatic purpose received before FOLFIRI-3*		
1 line only	52	80
2 lines or more	13	20
		
*FOLFOX regimen received before FOLFIRI-3*		
FOLFOX7	27	42
FOLFOX6	13	20
FOLFOX4	25	38

**Table 2:** Toxicity of FOLFIRI-3 according to NCI-CTC grading (maximal grade per patient, collected from 463 cycles, 65 patients)

	**NCI-CTC grade (% of patients)**			
	**1**	**2**	**3**	**4**
*Nonhaematological toxicities*				
Nausea	28	29	9	0
Vomiting	12	19	8	0
Mucositis	20	19	5	2
Diarrhoea	25	38	22	1
Asthenia	28	20	11	3
Hand–foot syndrome	23	2	0	—
Alopecia	23	54	—	—
				
*Haematological toxicities*				
Neutropenia	14	28	26	11
Febrile neutropenia	—	—	2	3
Anaemia	54	26	2	2
Thrombocytopenia	34	5	3	0

NCI-CTC, National Cancer Institute-Common Toxicity Criteria.

**Table 3:** RECIST number of the responder patients

**Patient no.**	**Response**	**RECIST at baseline**	**RECIST at first evaluation**	**RECIST at second evaluation**
103	PR	38	18	20
105	PR	10	6	3
108	PR	275	230	175
112	PR	114	45	50
113	PR	102	81	25
202	PR	90	90	65
212	PR	78	48	55
205	PR	20	10	10
301	PR	74	48	91
306	PR	80	70	44
308	PR	180	160	118
501	PR	100	90	70
505	PR	50	21	19
803	PR	131	90	78
404	CR	85	35	0

RECIST, Response Evaluation Criteria in Solid Tumors.

**Table 4:** Results of main published phase II and III studies of irinotecan in previously treated patients with metastatic colorectal cancer

**Reference**	**Study phase**	***N***	**Response rate (%)**	**Progression-free survival (months)**
*Irinotecan alone after 5FU*				
Van Cutsem *et al*, 1999	II	107	13.7	4
Pitot *et al*, 1997	II	90	13.3	—
Rothenberg *et al*, 1999	II	166	11	—
Rougier *et al*, 1997	II	140	16	—
Cunningham *et al*, 1998	III	189	—	—
Rougier *et al*, 1998	III	133	5	4.2
Fuchs *et al*, 2003	III	291	—	3.5
				
*Irinotecan combined with LV5FU after 5FU*				
Ducreux *et al*, 1999	II	55	22	6.3
Gil-Delgado *et al*, 2001	II	39	21	—
Rougier *et al*, 2002	Randomised phase II	101	11.4	—
				
*Irinotecan combined with LV5FU after FOLFOX*				
André *et al*, 1999	II	33	5.5	4.1
Tournigand *et al*, 2004	III	66	4	2.5
Mabro *et al*, 2003	II	29	17	4
Maindrault *et al*, 2002	II	20	20	6.7
FOLFIRI-3	II	65	23	4.7

## References

[bib1] André T, Louvet C, Maindrault-Goebel F, Couteau C, Mabro M, Lotz JP, Gilles-Amar V, Krulik M, Carola E, Izrael V, de Gramont A (1999) CPT-11 (irinotecan) addition to bimonthly, high-dose leucovorin and continuous-infusion 5-fluorouracil (FOLFIRI) for pretreated metastatic colorectal cancer. Eur J Cancer 35: 1343–1347 10658525 10.1016/s0959-8049(99)00150-1

[bib2] Cunningham D, Pyrhönen S, James RD, Punt CJ, Hickish TF, Heikkila R, Johannesen TB, Starkhammar H, Topham CA, Awad L, Jacques C, Herait P (1998) Randomised trial of irinotecan plus supportive care *vs* supportive care alone after fluorouracil failure for patients with metastatic colorectal cancer. Lancet 352: 1413–1418 9807987 10.1016/S0140-6736(98)02309-5

[bib3] de Gramont A, Figer A, Seymour M, Homerin M, Hmissi A, Cassidy J, Boni C, Cortes-Funes H, Cervantes A, Freyer G, Papamichael D, Le Bail N, Louvet C, Hendler D, de Braud F, Wilson C, Morvan F, Bonetti A (2000) Leucovorin and fluorouracil with or without oxaliplatin as first-line treatment in advanced colorectal cancer. J Clin Oncol 18: 2938–2947 10944126 10.1200/JCO.2000.18.16.2938

[bib4] Douillard JY, Cunningham D, Roth AD, Navarro M, James RD, Karasek P, Jandik P, Iveson T, Carmichael J, Alakl M, Gruia G, Awad L, Rougier P (2000) Irinotecan combined with fluorouracil compared with fluorouracil alone as first-line treatment for metastatic colorectal cancer: a multicentre randomised trial. Lancet 355: 1041–1047 10744089 10.1016/s0140-6736(00)02034-1

[bib5] Ducreux M, Ychou M, Seitz JF, Bonnay M, Bexon A, Armand JP, Mahjoubi M, Mery-Mignard D, Rougier P (1999) Irinotecan combined with bolus fluorouracil, continuous infusion fluorouracil, and high-dose leucovorin every two weeks (LV5FU2 regimen): a clinical dose finding and pharmacokinetic study in patients with pretreated metastatic colorectal cancer. J Clin Oncol 17: 2901–2908 10561369 10.1200/JCO.1999.17.9.2901

[bib6] Falcone A, di Paolo A, Masi G, Allegrini G, Danesi R, Lencioni M, Pfanner E, Comis S, Del Tacca M, Conte P (2001) Sequence effect of irinotecan on pharmacokinetics and toxicity in chemotherapy-naive metastatic colorectal cancer patients. J Clin Oncol 19: 3456–3462 11481350 10.1200/JCO.2001.19.15.3456

[bib7] Fuchs CS, Moore MR, Harker G, Villa L, Rinaldi D, Hecht J (2003) Phase III comparison of two irinotecan dosing regimens in second-line therapy of metastatic colorectal cancer. J Clin Oncol 21: 807–814 12610178 10.1200/JCO.2003.08.058

[bib8] Gil-Delgado MA, Guinet F, Castaing D, Adam R, Coeffic D, Durrani AK, Bismuth H, Khayat D (2001) Prospective phase II trial of irinotecan, 5-fluorouacil, and leucovorin in combination as salvage therapy for advanced colorectal cancer. Am J Clin Oncol 24: 101–105 11232943 10.1097/00000421-200102000-00021

[bib9] Goldberg RM, Sargent DJ, Morton RF, Fuchs CS, Ramanathan RK, Williamson SK, Findlay BP, Pitot HC, Alberts SR (2004) A randomised controlled trial of fluorouracil plus leucovorin, irinotecan, and oxaliplatin combinations in patients with previously untreated metastatic colorectal cancer. J Clin Oncol 22: 4–6 14665611 10.1200/JCO.2004.09.046

[bib10] Grothey A, Deschler B, Kroening H, Ridwelski K, Reichardt P, Kretzschmar A, Clemens M, Hirschmann W, Lorenz M, Asperger W, Buechele T, Schmoll HJ (2002) Phase III study of bolus 5-fluorouracil (5FU)/folinic acid (FA) (Mayo) *vs* weekly high-dose 24 h 5-FU infusion/FA+oxaliplatin (OXA) in advanced colorectal cancer (ACRC). Proc Am Soc Clin Oncol (Orlando, FL, USA) 21: 129a (abstract)

[bib11] Guichard S, Cussac D, Hennebelle I, Bugat R, Canal P (1997) Sequence-dependent activity of the irinotecan-5FU combination in human colon-cancer model HT-29 *in vitro* and *in vivo*. Int J Cancer 73: 729–734 9398054 10.1002/(sici)1097-0215(19971127)73:5<729::aid-ijc20>3.0.co;2-#

[bib12] Kaplan EL, Meier P (1958) Nonparametric estimation from incomplete observations. J Am Stat Assoc 53: 457–481

[bib13] Mabro M, Louvet C, André T, Carola E, Gilles-Amar V, Artru P, Krulik M, de Gramont A, on behalf GERCOR (2003) Bimonthly leucovorin, infusion, 5-fluorouracil, hydroxyurea, and irinotecan (FOLFIRI-2) for pretreated metastatic colorectal cancer. Am J Clin Oncol 25: 254–258 10.1097/01.COC.0000020581.59835.7A 12796595

[bib14] MacDonald J, Haller D, Mayer R (1995) Grading of toxicity. In: Manual of Oncologic Therapeutics, MacDonald J, Haller D, Mayer R (eds) pp 519–523. Lippincott: Philadelphia

[bib15] Maindrault F, Louvet C, Tournigand C, Gervais H, Mabro M, Artru P, Garcia ML, André T, Carola C, de Gramont A (2002) Leucovorin, 5-fluorouracil infusion and irinotecan (FOLFIRI-3) in pretreated patients with metastatic colorectal cancer. Proc Am Soc Clin Oncol (Orlando, FL, USA) 21: 658a (abstract)

[bib16] Mans DRA, Grivicich I, Peters GJ, Schwartsmann G (1999) Sequence-dependent growth inhibition and DNA damage by the irinotecan–5-fluorouracil combination in human colon carcinoma cell lines. Eur J Cancer 35: 1851–1861 10674003 10.1016/s0959-8049(99)00222-1

[bib17] Mullany S, Svingen PA, Kaufmann SH, Erlichman C (1998) Effect of adding the topoisomerase I poison 7-ethyl 10-hydroxycamptothecin (SN 38) to 5-fluorouracil and folinic acid in HCT8 cells: elevated dTTP pools and enhanced cytotoxicity. Cancer Chemother Pharm 42: 391–399 10.1007/s002800050835 9771954

[bib18] Pitot HC, Wender DB, O'Connell MJ, Schroeder G, Goldberg RM, Rubin J, Mailliard JA, Knost JA, Ghosh C, Kirschling RJ, Levitt R, Windschitl HE (1997) Phase II trial of irinotecan in patients with metastatic colorectal carcinoma. J Clin Oncol 15: 2910–2919 9256135 10.1200/JCO.1997.15.8.2910

[bib19] Rothenberg ML, Cox JV, DeVore RF, Hainsworth JD, Pazdur R, Rivkin SE, Macdonald JS, Geyer Jr CE, Sandbach J, Wolf DL, Mohrland JS, Elfring GL, Miller LL, Von Hoff DD (1999) A multicenter, phase II trial of weekly irinotecan (CPT-11) in patients with previously treated colorectal carcinoma. Cancer 85: 786–795 10091755

[bib20] Rougier P, Bugat R, Douillard JY, Culine S, Suc E, Brunet P, Becouarn Y, Ychou M, Marty M, Extra JM, Bonneterre J, Adenis A, Seitz JF, Ganem G, Namer M, Conroy T, Negrier S, Merrouche Y, Burki F, Mousseau M, Herait P, Mahjoubi M (1997) Phase II study of irinotecan in the treatment of advanced colorectal cancer in chemotherapy naive patients and patients pretreated with fluorouracil based chemotherapy. J Clin Oncol 15: 251–260 8996150 10.1200/JCO.1997.15.1.251

[bib21] Rougier P, Lepille D, Bennouna J, Marre A, Ducreux M, Mignot L, Hua A, Mery-Mignard D (2002) Antitumour activity of three second-line treatment combinations in patients with metastatic colorectal cancer after optimal 5-FU regimen failure: a randomised, multicentre phase II study. Ann Oncol 13: 1558–1567 12377643 10.1093/annonc/mdf259

[bib22] Rougier P, Van Cutsem E, Bajetta E, Niederle N, Possinger K, Labianca R, Navarro M, Morant R, Bleiberg H, Wils J, Awad L, Herait P, Jacques C (1998) Randomised trial of irinotecan *vs* fluorouracil by continuous infusion after fluorouracil failure in patients with metastatic colorectal cancer. Lancet 352: 1407–1412 9807986 10.1016/S0140-6736(98)03085-2

[bib23] Rowland KM, Pitot HC, Sargent DJ, Philip PA, Mitchell EP, Mailliard JA, Goldberg RM, Alberts SR (2005) Results of third line therapy on N9841: a randomized phase III trial of oxaliplatin/5-fluorouracil/leucovorin (FOLFOX4) *vs* irinotecan (CPT-11) in patients with advanced colorectal cancer previously treated with prior 5FU chemotherapy. Proc Am Soc Clin Oncol (Orlando, FL, USA) 24: 3519a (abstract)

[bib24] Saltz LB, Cox JV, Blanke C, Rosen LS, Fehrenbacher L, Moore MJ, Maroun JA, Ackland SP, Locker PK, Pirotta N, Elfring GL, Miller LL (2000) Irinotecan plus fluorouracil and leucovorin for metastatic colorectal cancer. Irinotecan study group. N Engl J Med 343: 905–914 11006366 10.1056/NEJM200009283431302

[bib25] Seymour MT, for the UK NCRI Colorectal Clinical Studies Group and FOCUS Trial Investigators (2005) Fluorouracil, oxaliplatin and CPT-11 (irinotecan), use and sequencing (MRC FOCUS): a 2135-patient randomized trial in advanced colorectal cancer (ACRC). Proc Am Soc Clin Oncol (Orlando, FL, USA) 24: 3518a (abstract)

[bib26] Therasse P, Arbuck SG, Eisenhauer EA, Wanders J, Kaplan RS, Rubinstein L, Verweij J, Van Glabbeke M, van Oosterom AT, Christian MC, Gwyther SG (2000) New guidelines to establish response to treatment in solid tumors. J Nat Cancer Inst 92: 205–216 10655437 10.1093/jnci/92.3.205

[bib27] Tournigand C, André T, Achille E, Lledo G, Flesh M, Mery-Mignard D, Quinaux E, Couteau C, Buyse M, Ganem G, Landi B, Colin P, Louvet C, de Gramont A (2004) FOLFIRI followed by FOLFOX6 or the reverse sequence in advanced colorectal cancer: a randomized GERCOR study. J Clin Oncol 22: 229–237 14657227 10.1200/JCO.2004.05.113

[bib28] Van Cutsem E, Cunningham D, Ten Bokkel Huinink WW, Punt CJ, Alexopoulos CG, Dirix L, Symann M, Blijham GH, Cholet P, Fillet G, Van Groeningen C, Vannetzel JM, Levi F, Panagos G, Unger C, Wils J, Cote C, Blanc C, Herait P, Bleiberg H (1999) Clinical activity and benefit of irinotecan (CPT-11) in patients with colorectal cancer truly resistant to 5-fluorouracil (5-FU). Eur J Cancer 35: 54–59 10211088 10.1016/s0959-8049(98)00353-0

